# A *de novo* genome assembly and annotation of the southern flying squirrel (*Glaucomys volans*)

**DOI:** 10.1093/g3journal/jkab373

**Published:** 2021-11-12

**Authors:** Jesse F Wolf, Jeff Bowman, Sonesinh Keobouasone, Rebecca S Taylor, Paul J Wilson

**Affiliations:** 1 Biology Department, Trent University, Peterborough, ON K9J 7B8, Canada; 2 Ontario Ministry of Northern Development, Mines, Natural Resources and Forestry, Wildlife Research and Monitoring Section, Trent University, Peterborough, ON K9J 7B8, Canada; 3 Landscape Science and Technology Division, Environment and Climate Change Canada, Ottawa, ON K1S 5R1, Canada

**Keywords:** Northern flying squirrel, Southern flying squirrel, *Glaucomys volans*, *Glaucomys sabrinus*, hybrid zone, introgression, comparative genomics

## Abstract

Northern (*Glaucomys sabrinus*) and southern (*Glaucomys volans*) flying squirrels are widespread species distributed across North America. Northern flying squirrels are common inhabitants of the boreal forest, also occurring in coniferous forest remnants farther south, whereas the southern flying squirrel range is centered in eastern temperate woodlands. These two flying squirrel species exhibit a hybrid zone across a latitudinal gradient in an area of recent secondary contact. *Glaucomys* hybrid offspring are viable and can successfully backcross with either parental species, however, the fitness implications of such events are currently unknown. Some populations of *G. sabrinus* are endangered, and thus, interspecific hybridization is a key conservation concern in flying squirrels. To provide a resource for future studies to evaluate hybridization and possible introgression, we sequenced and assembled a *de novo* long-read genome from a *G. volans* individual sampled in southern Ontario, Canada, while four short-read genomes (two *G. sabrinus* and two *G. volans*, all from Ontario) were resequenced on Illumina platforms. The final genome assembly consisted of approximately 2.40 Gb with a scaffold N50 of 455.26 Kb. Benchmarking Universal Single-Copy Orthologs reconstructed 3,742 (91.2%) complete mammalian genes and genome annotation using RNA-Seq identified the locations of 19,124 protein-coding genes. The four short-read individuals were aligned to our reference genome to investigate the demographic history of the two species. A principal component analysis clearly separated resequenced individuals, while inferring population size history using the Pairwise Sequentially Markovian Coalescent model noted an approximate species split 1 million years ago, and a single, possibly recently introgressed individual.

## Introduction 

High-throughput sequencing studies on hybrid zones of wild non-model species have revealed traits associated with divergence in sympatry and allopatry (Scordato *et al.* 2017), patterns of introgression that differ between populations (Nolte *et al.* 2009), and genes associated with reproductive isolation (Teeter *et al.* 2008). Whole-genome sequencing provides insight into the evolutionary process of hybridization and adaptive introgression, however, demonstrating the adaptive or fitness values of introgressed genomic regions remains an area of difficulty (Taylor and Larson 2019). Studies of this kind benefit from a reference genome as a basis for identifying genomic regions of interest, and against which it is possible to evaluate potential hybrids and introgressed individuals (Payseur and Rieseberg 2016).

Hybridization and introgression can occur between closely related species brought into secondary contact (Chown *et al.* 2015). An increase in global surface temperatures has led to range shifts among a variety of taxa on a global scale (Chen *et al.* 2011) and increasing secondary contact between closely related species (Krosby *et al.* 2015), leading to increased opportunities for hybridization (Garroway *et al.* 2010; Chunco 2014). Hybridization can be an evolutionary dead end, or it can lead to adaptive introgression (Arnold and Martin 2009; Abbott *et al.* 2013). Introgression can result in the merging of hybridizing forms, reinforcement of reproductive barriers through selection for assortative mating, and a non-neutral shift in fitness among introgressed individuals. In some instances, this enables the expansion of the introgressed species into a novel habitat (Arnold 1992). Further complicating this, adaptive introgression combined with climate change can weaken reproductive isolation (Owens and Samuk 2020). In its extreme form, hybridization can drive extinction through introgression (Rhymer and Simberloff 1996).

Climate-driven range expansions have been noted in mammals, insects, and fish (Moritz *et al.* 2008; Garroway *et al.* 2010; Muhlfeld *et al.* 2014; Scriber 2014), among other taxa. Instances of hybridization in wild ecosystems can be exacerbated by climate change because of increased secondary contact, where barriers to interspecific reproduction are reduced or removed altogether (Chunco 2014). Without such barriers, species that were previously allopatric might interbreed, possibly leading to genetic admixture and outbreeding depression or heterosis (Barton 2001; Rius and Darling 2014).

As climate-mediated range expansion has been shown to increase distributional overlap between related species (Chunco 2014), climate change will therefore likely drive interspecific hybridization between many taxa. For example, studies in North America have noted hybrid zones across a latitudinal gradient between southern (*Glaucomys volans*) and northern (*Glaucomys sabrinus*) flying squirrels (Garroway *et al.* 2010; Rogic *et al.* 2016). Interspecific hybridization is a key conservation concern for these flying squirrel species, as population declines among northern flying squirrels have been noted in some areas of the United States, where some populations are endangered (Wood *et al.* 2016). The potential for introgressive hybridization and the subsequent ecological and fitness consequences necessitates a holistic assessment of species biology in the *Glaucomys* hybrid zone. The hybrid zone can be a valuable study system to facilitate the assessment of interspecific hybridization, the potential for reinforcement of reproductive barriers, and the associated ecological conclusions in a wild, *in* *vivo* system.

Low hybrid fitness can also lead to increased divergence between species through reinforcement. *Glaucomys* hybrid offspring are viable and can successfully backcross with either parental species (Garroway *et al.* 2010), however, the fitness implications among hybrid or introgressed individuals is unknown. The purpose of our study was to generate a *de novo* reference genome for *Glaucomys* as a basis for identifying genomic regions of interest and to aid in evaluation of potential hybrids and introgressed individuals in future research. We annotated the reference genome using our already assembled and annotated flying squirrel transcriptome (Brown *et al.*, 2021). Subsequently, using short reads from four individuals, two northern and two southern flying squirrels, we assembled re-sequenced high coverage genomes by aligning to the reference genome for a comparative analysis and demographic history reconstruction.

## Materials and methods

### Sample preparation

**Figure 1:**
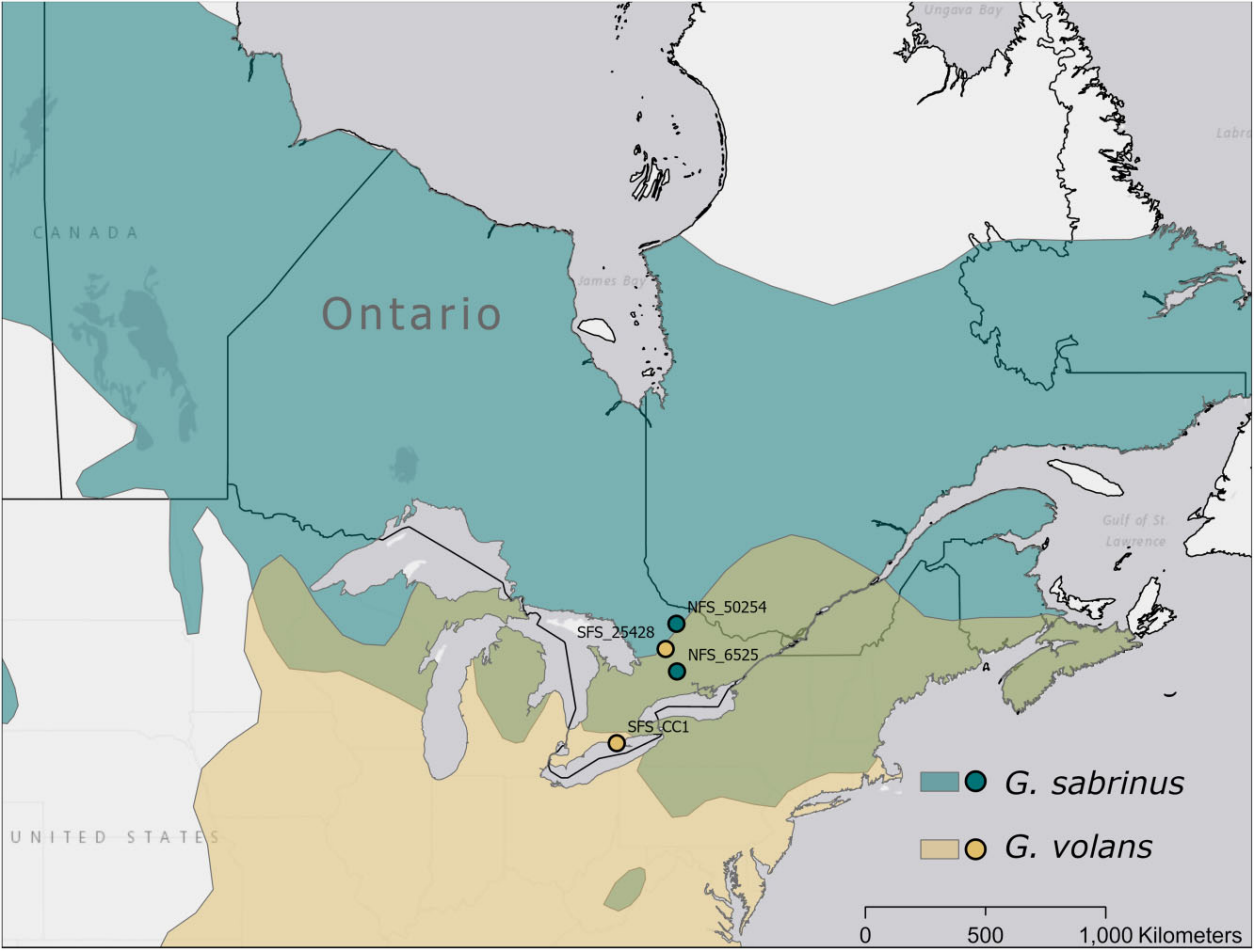
Range of northern (*Glaucomys sabrinus*) and southern (*Glaucomys volans*) flying squirrels as delineated by IUCN (Cassola 2016a, 2016b), overlaid with sampling locations. The geographic ranges are represented in the same colors as samples, while the hybrid zone is represented in olive. Both the northernmost *G. sabrinus* sample from Algonquin Provincial Park, Ontario, and the southernmost *G. volans* sample from Clear Creek, Ontario, were located outside of the hybrid zone. The southernmost *G. volans* sample (SFS_CC1) was used for *de novo* assembly.

We isolated brain tissue from two adult *G.* *volans* and two adult *G.* *sabrinus* for sequencing. Approximately 1.0 g of frozen brain tissue was removed from the hindbrain of each individual and immediately stored in RNAlater-ICE (see Brown *et al.*, 2021 for additional details). *Glaucomys sabrinus* individuals were collected from near Kawartha Highlands Signature Site Park (Northern Flying Squirrel 6525; NFS_6525, female; 44.689°N, 78.335°W) and in Algonquin Provincial Park, ON, Canada (Northern Flying Squirrel 50254; NFS_50254, male; 45.583°N, 78.466°W), and *G. volans* individuals were sampled near Sherborne Lake (Southern Flying Squirrel 25428; SFS_25428, male; 45.179°N, 78.840°W) and Clear Creek, Ontario, Canada (Southern Flying Squirrel CC1; SFS_CC1, female; 42.523°N, 81.628°W; Figure  1). Algonquin Provincial Park (NFS_50254) was outside the northern edge of the hybrid zone, and Clear Creek (SFS_CC1) was outside the range of *G. sabrinus* and not an area of sympatry. The sites were all mature, closed canopy forests with a mixture of temperate deciduous trees such as sugar maple (*Acer saccharum*), red oak (*Quercus rubra*), and American beech (*Fagus grandifolia*), and coniferous trees such as white pine (*Pinus strobus*) in uplands or white spruce (*Picea glauca*) and balsam fir (*Abies balsamea*) in riparian areas (see Bowman *et al.* 2005 for more details). All four specimens were morphologically identified to their parental species. Squirrel tissue samples were extracted using a phenol-chloroform extraction. The extracted DNA was run on a 1.5% agarose gel and Qubit fluorometer using the High Sensitivity Assay Kit to ensure we had sufficient DNA. The DNA extractions were also run on a Nanodrop ND-8000 spectrophotometer to test purity. The DNA was normalized to 20 ng/µl at a final volume of 50 µl.

### *De novo* genome assembly

Southern flying squirrel libraries from individual SFS_CC1 were prepared and paired-end sequenced on 1 lane on an Illumina HiSeq X to generate 150 base pair (bp) paired-end reads. Sequencing was conducted at The Centre for Applied Genomics (Next Generation Sequencing Facility, SickKids Hospital, Toronto, ON, Canada). The sequence reads from each sample were provided in a FASTQ file format. 10X Genomics long read Chromium sequencing was used to generate linked reads. The estimated genome size was thought to be similar to that of the eastern grey squirrel (*Sciurus carolinensis*) genome (2.82 Gb; Mead *et al.* 2020). We used FastQC (version 0.11.9; Andrews 2010) to perform simple quality control checks on raw sequence data to confirm the quality of the trimmed sequence reads. Long reads were assembled using Supernova as this assembler uses 10X linked-reads to produce phased assemblies of homologous chromosomes over multi-megabase ranges (Weisenfeld *et al.* 2018). The FASTA file representing the assembly was generated using the pseudohap style output. Assembly statistics were generated using BBMap 38.90 (Bushnell *et al.* 2017). We used BUSCO v 3.1.0 (Benchmarking Universal Single-Copy Orthologs; Waterhouse *et al.* 2018) and the mammalia odb9 dataset to reconstruct 4,104 conserved mammalian genes to assess genome completeness.

### Resequenced genome assemblies

Northern and southern flying squirrel libraries were prepared and paired-end sequenced across eight lanes on an Illumina HiSeq X to generate 150 bp paired-end reads. Sequencing was conducted at The Centre for Applied Genomics (Next Generation Sequencing Facility, SickKids Hospital, Toronto, ON, Canada). Forward and reverse reads were concatenated across eight lanes. FastQC was run as above to determine forward and reverse read quality and inform subsequent trimming parameters. We trimmed the adapters and low-quality bases from the reads with Trimmomatic v0.39 and parameter specifications as follows: Illumina adapters were removed, leading and trailing low quality or N bases were removed (below quality 3), reads were scanned with a 4-base sliding window and cut when the average per quality base drops below 15, and reads were dropped that were less than 36 bases long after the previous step (Bolger *et al.* 2014). To avoid any potential contamination of the genome sequence with viral or bacterial sequences, we screened the trimmed reads with Kraken2 (Wood *et al.*, 2019) using the full standard database.

Reads from four individuals, including the individual used for the *de novo* assembly, (NFS_6525, NFS_50254, SFS_25428, and SFS_CC1) were aligned to the synthetic linked-read reference genome using Bowtie2 2.2.4 (Langmead and Salzberg 2012), and the SAM file was converted to a BAM file using Samtools 1.7 (Li *et al.* 2009). We removed poorly mapped reads via skipping alignments with MAPQ values smaller than 20 using Samtools 1.7. We removed duplicate reads and added correct read group information to each BAM file using Picard 2.18.27 (http://broadinstitute.github.io/picard/). We then clipped overlapping regions using clipOverlap from bamUtil 1.0.1.4 (Jun *et al.* 2015) and sorted the BAM file using Samtools 1.7 and built an index using Picard. All BAM files were checked using FastQC 0.11.9 (Andrews 2010), and we calculated the mean depth of coverage for each BAM file using Samtools. We used Haplotype Caller in gatk 3.8 (Mckenna *et al.* 2010) to call variants and produce a variant call format (VCF) file for each flying squirrel. Individual VCF files were combined using the Combine GVCFs function, and then, we performed joint genotyping using Genotype GVCFs, both in GATK, to produce a VCF file with both northern and southern flying squirrels. We did some additional filtering on the combined VCF files to ensure quality. We used VCFtools 0.1.16 (Danecek *et al.* 2011) to do two rounds of filtering. First, we removed indels (using the remove-indels command), and any site with a depth of less than five or more than 33 (approximately double the average depth across the genome, using the min-meanDP and max-meanDP commands) and removed any low-quality genotype calls, with a score below 20 (using the minGQ command), which in VCFtools are changed to missing data. In the second round, we filtered to remove genotypes with more than 10% missing data (using the max-missing command). We did not filter to remove any SNP with a minor allele frequency (MAF) as we have only one individual from each location and this results in removing the private sites, instead relying on very high depth and stringent filtering to ensure a high-quality data set.

The combined VCF file used for analyses with all individuals contained 35,937,561 SNPs. After filtering, we measured the mean depth (using the depth command) and the frequency of missing data (using the missing-indv command) for each individual in the final VCF file of 2 northern and 2 southern flying squirrels using VCFtools.

### Annotation

We identified and classified the repeat regions of the assembled genome using RepeatMasker v. 4.1.0 (Smit *et al.* 2013). We configured RepeatMasker with the RMBlast v. 2.10.0 sequence search engine, Tandem Repeat Finder v. 4.0.9 (Benson, 1999), Dfam_Consensus database 3.1 (November 2020 release), and used the “-species glaucomys” parameter for the analysis.

We used the gene prediction program AUGUSTUS 2.5.5 (Hoff and Stanke 2019) to annotate the masked genome using predictions based on human genes. In addition, we incorporated RNA-Seq data into AUGUSTUS using the transcriptome created from brain tissue by Brown *et al*., (2021). We used BLAT v. 1.04 to help identify exon structure and allow for the subsequent generation of both intron and exon hints from alignments for AUGUSTUS (Hoff and Stanke 2019; http://augustus.gobics.de/binaries/readme.rnaseq.html). The genome run in AUGUSTUS used a partial gene model allowing the prediction of incomplete genes at the sequence boundaries. The masked genome was split into 31 parts of ∼1,995 sequences each to reduce the computational resources and we concatenated the 31 output general feature format (GFF) files into a single annotation file.

### Comparative analyses

To compare whole-genome heterozygosity estimates, we used ANGSD to generate a site frequency spectrum and obtain heterozygosity values for each individual. We used the parameters -C 50 -ref ref.fa -minQ 20 -minmapq 30 to remove the low-quality bases and reads (Korneliussen *et al.* 2014). We generated a principal component analysis (PCA) to determine the degree of differentiation between these samples. We also ran Pairwise Sequentially Markovian Coalescent (PSMC; Li and Durbin 2011) to model the historical effective population size and reconstruct the demographic history of both our northern and southern flying squirrel genomes. We used the default parameters of 64 atomic time intervals (-p “4 + 25*2 + 4 + 6”), a generation time of 1.5 years (COSEWIC 1998), and a mutation rate of *m* = 2.0 × 10^−9^ mutations/site/generation (Gossmann *et al.* 2019).

## Results and discussion

### *Glaucomys volans* genome assembly

The final *G.* *volans* genome assembly was the untrimmed linked-read 10X Chromium assembly with Supernova (Weisenfeld *et al.* 2018), which produced a genome consisting of 7,087 scaffolds ≥50 Kb, a scaffold N50 of 455.26 Kb, a contig N50 of 75.63 Kb, a GC content of 40.48%, and a genome size of 2.40 Gb (Tables  1 and 2). Although the genome produced here is fragmented compared to the Earth BioGenome goals, future flying squirrel research will benefit greatly from the presence of a reference genome. BUSCO indicated the presence of 3,742 (91.2%) complete mammalian genes of the 4,104 searched for. Our estimated genome size was similar to the assembly of the thirteen-lined ground squirrel (*Ictidomys tridecemlineatus*; ∼2.5 Gb), whereas the BUSCO value for the ground squirrel was 92.9% (Di Palma *et al.* 2011). Genome annotation of our final genome incorporating RNA-Seq data identified the locations of 19,124 protein-coding genes compared to 28,262 protein-coding genes without using RNA-Seq data.

**Table 1:** Summary statistics of the long read *Glaucomys volans* reference genome

Statistic	*Glaucomys volans* genome
Scaffold sequence total (bp)	2.58 × 10^9^
Number of scaffolds	61,815
Scaffold N50 (bp)	455,262
Scaffold L50	1,582
Scaffold N90 (bp)	117,214
Scaffold L90	5,080
Contig sequence total (bp)	2.53 × 10^9^
Number of contigs	115,069
Contig N50 (bp)	75,631
Contig L50	9,446
Contig N90 (bp)	21,155
Contig L90	30,374

**Table 2:** Nucleotide base composition of the long-read *Glaucomys volans* reference genome

A	C	T	G	N
29.77%	20.24%	29.75%	20.24%	0.17%

### Resequenced genome assembly

Trimming the concatenated short read pairs resulted in the removal of an average of 4.37% of reads. The human library was removed from the full standard database, as its inclusion resulted in a relatively high percentage of reads mapped as human due to orthologous mammal genes. After removing the human library, 0.25–0.35% of the reads were classified as belonging to an identified bacterial taxon; screening trimmed concatenated short read pairs for bacterial contaminants resulted in the further removal of an average of 0.29% of reads. The final short read coverage for each of the four individuals were as follows: SFS_CC1 = 15.75X, SFS_25428 = 17.55X, NFS_50254 = 17.88X, and NFS_6525 = 14.96X. Our final VCF file contained 10% missing data. For all individuals, observed heterozygosity exceeded expected, while inbreeding coefficients ranged from 0.00261 to 0.00358 (NFS_50254 = 0.00276, NFS_6525 = 0.00261, SFS_CC1 = 0.00311, and SFS_25428 = 0.00358).

### Comparative analyses and population history of *G. sabrinus and G. volans*

**Figure 2:**
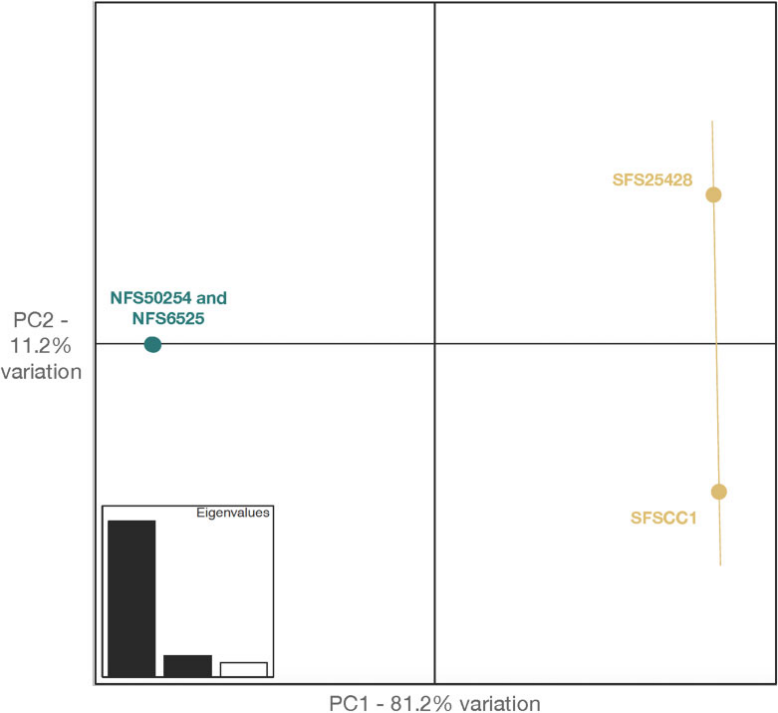
PCA of two northern (*G. sabrinus—*represented in turquoise) and two southern (*G. volans—*represented in yellow) flying squirrel genomic variation. PC1 (*x*-axis) accounts for 81.2% of the variation, while PC2 (*y*-axis) accounts for 11.2% of the variation; combined, the first two principal components account for over 90% of the genomic variation.

Northern and southern flying squirrels grouped distinctly in our PCA, while there was more variation among southern flying squirrels, possibly due to collection locations that were farther apart (Figure  2). The first principal component accounted for over 80% of the variation noted, and clearly separated both species. Both southern individuals had higher whole-genome heterozygosity relative to northern individuals. There are multiple possible explanations for this result. For example, southern flying squirrels are smaller-bodied and typically exhibit higher population sizes and densities, whereas a lower effective population size in northern flying squirrels may result in decreased heterozygosity (Arbogast 2007; Bowman *et al.* 2020). Overall, the levels of heterozygosity of both flying squirrel species that we observed were comparable to other genome-wide estimates in mammals (see Figure  3 in Morin *et al.* 2021).

**Figure 3:**
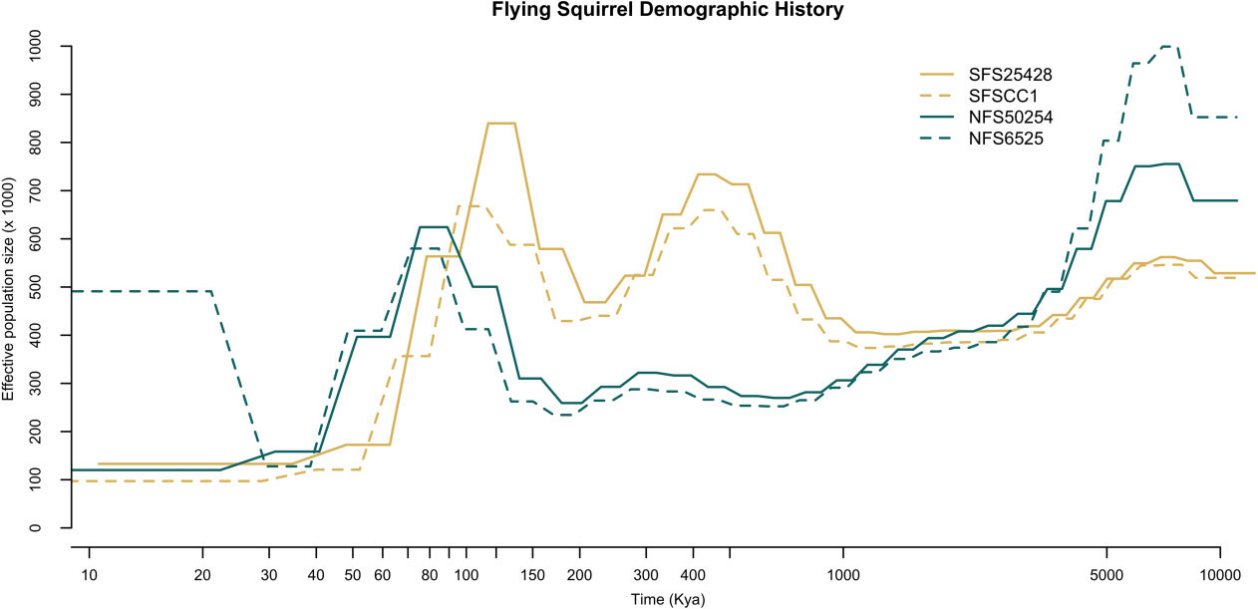
Reconstruction of historical effective population size (*N*_e_) of both northern (*G. sabrinus—*represented in turquoise) and southern (*G. volans—*represented in yellow) flying squirrels using PSMC analysis assuming a mutation rate µ of 2.0 × 10^−9^ mutations/site/generation and a generation time of 1.5 years. *N*_e_ is in units of 1,000 individuals on the *y*-axis and time measured in thousands of years ago (Kya) is on the *x*-axis. Dashed lines separate the individuals of the same species.

Previous research has estimated the split between northern and southern flying squirrels to be in the early to mid-Pleistocene (2,580,000–130,000 years ago; Arbogast 1999, 2007). Based on PSMC analysis, the split between the species seemed to occur approximately 1mya, whereas, after 1mya, the species exhibited different trajectories (Figure  3). The demographic reconstruction of one northern flying squirrel (NFS_6525) showed an increase in recent effective population size relative to the other northern individual (NFS_50254; Figure  3). It is possible that the demographic history of NFS_50254 more closely resembles that of the southern flying squirrel samples. However, it is also possible that technical biases resulted in this pattern (*e.g.*, relatively low sequencing coverage for NFS_6525), as PSMC is less accurate in the recent past and is susceptible to an increase in effective population size as seen in our data (*e.g.*, Nadachowska-Brzyska *et al.* 2016). Previous work using microsatellites has been consistent with panmixia in Ontario within each of these species (Garroway *et al.* 2011; Bowman *et al.* 2020). To address introgression and hybridization concerns, future research can utilize the reference genome produced here to perform analyses with a larger sample of genomes with varying degrees of introgression to help clarify these patterns.

## Conclusion

We produced a high-quality southern flying squirrel reference genome, an annotation in gff3 and bed format, and a RepeatMasked version of the genome, as well as high-coverage northern and southern flying squirrel re-sequenced genomes. The availability of a high-quality reference genome is invaluable in answering evolutionary questions surrounding hybridization and introgression and for conservation efforts. This is the first flying squirrel genome generated and will help future research determine not only the presence of hybrids in the North American flying squirrel hybrid zone but can also aid in identifying loci of interest in these same populations.

## Data availability

10X Chromium long-read and Illumina short-read data are available at the National Center for Biotechnology Information (NCBI), under the BioProject accession number PRJNA723586. This Whole Genome Shotgun project has been deposited at DDBJ/ENA/GenBankunder the accession JAJEJO000000000. The version described in this paper is version JAJEJO010000000. 
